# New Horizons in Cationic Photopolymerization

**DOI:** 10.3390/polym10020136

**Published:** 2018-01-31

**Authors:** Marco Sangermano, Ignazio Roppolo, Annalisa Chiappone

**Affiliations:** Politecnico di Torino, Dipartimento di Scienza Applicata e tecnologia, C.so Duca degli Abruzzi 24, 10129 Torino, Italy; ignazio.roppolo@polito.it (I.R.); annalisa.chiappone@iit.it (A.C.)

**Keywords:** cationic photopolymerization, cationic initiators, Radical-Induced Cationic Photopolymerization, Frontal Polymerization

## Abstract

In this review, we report some recent advances and new horizons in UV-induced cationic photopolymerization. In particular, after a brief introduction on the discovery and affirmation of the cationic photopolymerization process, new efforts in the synthesis of cationic photoinitiators are reported. Subsequently, an interesting and absolutely new application is reported, related to the combination of Radical-Induced Cationic Photopolymerization with Frontal Polymerization, achieving the cross-linking of epoxy composites.

## 1. Introduction

Light-induced polymerization reactions are being met with continuous growing interest in different fields, from protective coatings [1], to graphic arts [2], microelectronics [3], dental applications [4], security inks [5], and, finally, 3D-printing technologies [6,7].

This polymerization technique shows a number of economic advantages over the usual thermal operation, such as rapid curing, low energy requirements, room-temperature treatment, nonpolluting and solvent-free formulations, and low cost [8]. Among the different mechanisms, cationic photopolymerization is finding new deep interest in scientific literature. 

Cationic photo-induced polymerization was discovered by Prof. Crivello [9,10,11,12,13,14] in the late 70s when he found out that under UV irradiation, onium salts (triphenylsulfonium or diaryliodonium) generate a very acid solution, which was able to promote cationic polymerization of a vinyl monomer in the dark. 

“Anatomically” analyzing these salts, they are obviously composed of cationic and anionic moieties. The cationic side is the light-absorbing component and seat of the photochemistry; therefore, the structure of the cation controls the UV absorption characteristics, the photosensitivity, the quantum yield, whether the compound can be photosensitized or not, and the ultimate thermal stability of the compound. On the other hand, it is the nature of the anion that determines the strength of the acid formed during photolysis and its corresponding initiation efficiency. The nature of the anion also determines the character of the propagating ion pair. This has a direct impact on the kinetics of polymerization and the rate of termination [15]. In conclusion, these salts can be viewed as photoacid generators. In fact, the final photodecomposition product is a very strong Brønsted acid which is able to start the cationic chain growth polymerization.

The photodecomposition mechanism of the onium salts first involves photoexcitation and then the decay of the resulting excited singlet state, with both heterolytic and homolytic cleavages. The aryl cations and aryliodine cation radicals generated during photolysis are highly reactive species and further react with solvents, monomers, or impurities to give protonic acids, which are the predominant initiators of cationic polymerization [16,17,18]. 

**Scheme 1:**
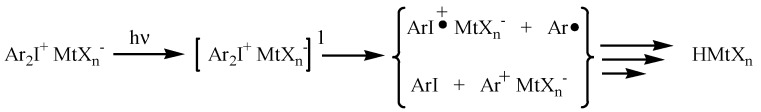
Representation of photodecomposition of a general diaryliodonium salt, where MtX is counteranion (typically SbF^−^_6_, PF^−^_6_, BF^−^_4_).

A simplified schematic representation of the photoactivation of a generic diaryliodonium salt is reported in Scheme 1.

As previously mentioned, the strength of the generated photoacid is directly related to the nature of the counteranion. The bigger is the counteranion, the lower is its nucelophilicity and, thus, the stronger is the related photogenerated acid [19]. 

One peculiar advantage of the cationic UV curing process is the absence of oxygen inhibition during polymerization, which is the main problem of the alternative radical-induced photopolymerization. This eliminates the need of an inert atmosphere during curing, distinguishing cationic from radical polymerization [20]. Furthermore, the monomers employed in cationic UV curing are characterized by an absence of toxicity or irritation characteristics, unlike the acrylate and methacrylate systems usually employed in radical processes: for this reason, these monomers are a preferable alternative. Last, but not least, a lower shrinkage after curing is observed in the cationic photopolymerization mechanism, generating lower residual stresses in the cured materials and inducing better adhesion properties on different substrates, with respect to the radical mechanism [21].

Cationic photopolymerization has been deeply reviewed in the past [8,13,14,22] but the aim of this paper is to review some very recent advancements in the field of cationic UV curing process—in particular, those related to the synthesis of new photoinitiators and the activation of frontal polymerization for the curing of thick systems. This could open new horizons in the use of cationic photopolymerization. 

## 2. New Cationic Photoinitiators

After the discovery of the “onium” salts by Crivello and their massive use in photopolymerization, little attention has been devoted to the synthesis of new photoinitiating systems. Some years ago, the limitations related to the activation wavelength of the photoinitators were deeply investigated by Yagci [23,24,25,26] and by Crivello himself [8,9,10,11,12,13]. 

In fact, by a chemical modification of the onium salts (introducing chromophoric groups on the aromatic rings), it is possible to shift the activation wavelength [27]. Alternatively, onium salts can be indirectly activated through electron transfer [28,29,30] or excitation of the charge-transfer complex of onium salts [31,32]. In these cases, thermodynamics controls the efficiency of photodecomposition. Typically, photosensitizers are conjugated aromatic hydrocarbons, characterized by a low oxidation potential, so that they are able to activate the photosensitization of conventional onium salts [33].

At last, it is possible to activate the photodecomposition of a cationic photoinitiator to longer wavelengths by the oxidation of free radicals through a radical-induced cationic photopolymerization mechanism [34,35]: in the presence of reactive free radical species and of an appropriate onium salt, an oxidation reaction is promoted with the transformation of a carbon center radical to a carbocationic species—one that is reactive enough to promote the cationic chain growth polymerization reaction [36,37,38].

Since 2011, many novel photoinitiating systems based on organic and organometallic compounds with excellent visible light absorption have emerged exhibiting outstanding photoinitiating abilities, especially for cationic photopolymerization [39]; also, organic dyes and even silanes have been proposed to activate the cationic photopolymerization process by using a longer wavelength. A very recent review reports detailed information on the state of the art of photoinitiating systems for cationic polymerization [40]. 

**Figure 1:**
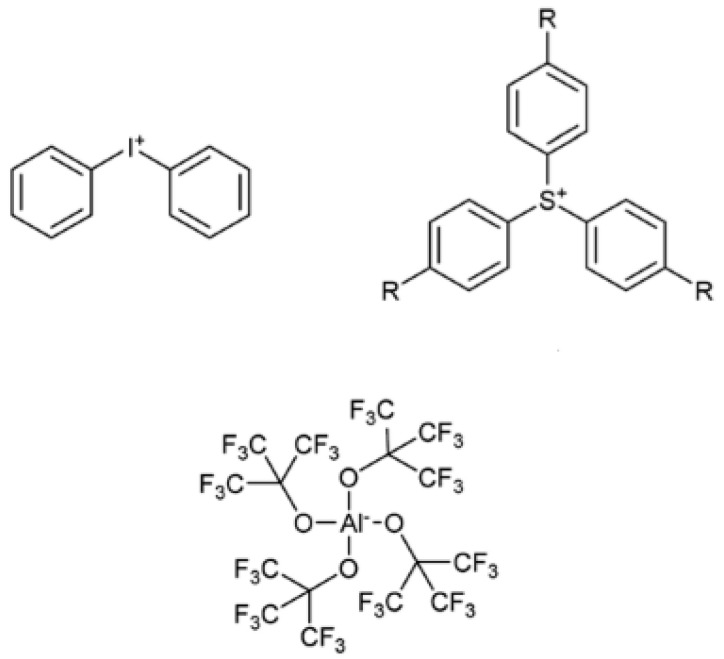
Schematic representation of iodonium or sulfonium salts containing tetrakis(perfluoro-*t*-butyloxy)aluminate anion.

Klikovits et al. proposed the synthesis of a new cationic photoinitiator based on the tetrakis(perfluoro-*t*-butyloxy)aluminate anion. In Figure 1, the schematic representation of iodonium or sulfonium salts are reported with the alluminate counter ion. The reactivity of these new cationic photoinitiators compared with that of common onium salt was studied by using photo-DSC, showing the advantageous reactivity of the novel alkoxyaluminate-based cationic compounds [41]. 

The photo-DSC studies revealed the higher reactivity of the novel photoinitiator in comparison to that of commercially available photoinitiators, and, in particular, showed a much higher ability to initiate cationic polymerization of the bisphenol-A-diglycidyl ether monomer. More importantly, the alluminated photoinitiator showed very good results in photosensitization.

**Figure 2:**
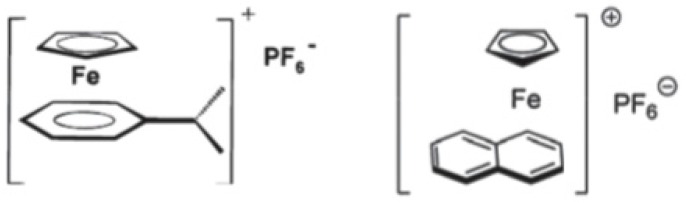
Schematic representation of some ferrocenium salts.

Recently, Zhang et al. reported on the use of ferrocenium salts as new cationic photoinitiators, which exhibit the broadest visible light absorption available given the state of the art [42]. In Figure 2, the chemical structures of some of these salts are reported.

These new photoinitiators have been proposed to promote the cationic polymerization of epoxides through photoredox catalysis processes upon exposure to near-UV (385 nm) or visible violet (405 nm) light-emitting diodes (LEDs). The authors showed that by combining the iron complex-based photoinitiating systems with an iodonium salt and *N*-vinylcarbazole, generation of radicals, cations, and radical cations occurs, providing evidence of good efficiency in polymerizing cationic photocurable monomers [43].

As an alternative to ferrocenium salts, the same authors proposed the use of a highly porous iron(III)-based metal–organic framework (MOF) that in the presence of an iodonium salt and *N*-vinylcarbazole is able to initiate the free-radical-promoted cationic polymerization of epoxides upon exposure to near-UV (385 nm) or visible (405 nm) light-emitting diodes in a standard air environment [44]. In Table 1, some proposed MOF structures are reported along with their main properties.

Very recently there have been reports in the literature about dual-cure initiating systems based on pyrylium salts [45]. The molecule 2,4,6-triphenylpyrylium tetrafluoroborate behaves as an efficient cationic photo- and thermal initiator. Its absorption in the UVA–visible range allows the photopolymerization of epoxy resins under 395 nm LEDs. Furthermore, surface photopolymerization with LEDs was used to activate a thermal polymerization of the resin in depth through a photo-induced thermal frontal polymerization process. This opens the opportunity to develop new initiating systems for photo-induced polymerization of thick and filled epoxy resins: This will be the topic of this review in the next paragraph.

Photo-induced living cationic polymerization of isobutyl vinyl ether were achieved in the presence of various combinations of halides of diphenyliodonium and zinc salts [46].

Lecompère and coworkers investigated the effect of the presence of additives such as hydrogen peroxides or vinyl ethers on the polymerization of thick samples. By using 1 wt % of hydrogen peroxide or 3 wt % of isobutylvinylether, the authors showed the possibility to perform a photo-induced thermal frontal polymerization at 395 nm up to a depth of 3 cm. When hydrogen peroxide was added, an average polymerization velocity of 1.1 cm/min was measured, while 2.6 cm/min in the presence of isobutylvinylether was achieved. By increasing the amount of coinitiator, the energy required to support the front of polymerization decreases, leading to a rise in the velocity. This effect could be a great advantage when the formulation contains fillers to promote the displacement of the front.

## 3. Frontal Polymerization

**Scheme 2:**
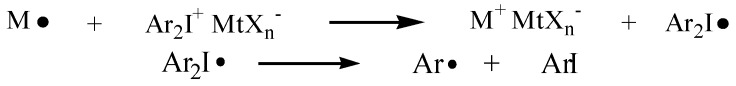
Representation of the mechanism of redox nonradiative activation of iodonium salt.

It is well known that the main limitation of photocuring is related to the processable thickness of the layer because of the low penetration depth of UV light [47,48]. On the other hand, it is well documented in literature that the decomposition of onium salt can be induced either by irradiation with UV light or via a redox reaction in the presence of suitable radicals. This nonradiative decomposition process of onium salt was called radical-induced cationic polymerization (RICP) by Crivello [49,50]. Iodonium salts can be reduced to diaryliodine radicals in the presence of a carbon radical, which is oxidized to a carbocation. In a following reaction, the diaryliodine radical decays to aryliodide and an aryl free radical. Since the counterion of the redox-generated carbocation has low nucleophilicity, the carbocation is reactive enough to promote ring-opening polymerization (see Scheme 2). 

In other words, a free radical chain reaction is promoted in which the diaryliodonium salt photoinitiator is consumed by a non-photochemical process; as a consequence, reactive carbocations are generated.

A coupling of RICP with frontal polymerization (FP) has been suggested in the literature. FP is a reaction that, after local initiation by a proper stimulus, can proceed to adjacent zones by the movement of a reaction front [48]. The propagation of the FP derives from the thermal decomposition of suitable initiators: the required heat to activate the initiators comes from the hexotermicity of the occurring polymerization. Therefore, it is possible to couple RICP with FP. In particular, FP can occur through the dissociation of a radical thermal initiator promoted by the heat released during surface UV-induced cationic polymerization. The thermal generated radicals are oxidized to carbocations by the presence of iodonium salt. If heat dissipation is not too high, a hot polymerization front capable of self-sustaining is generated and the RICP proceeds into the deeper layers of the formulation. 

This complex mechanism can be called radical-induced cation frontal polymerization (RICFP); with controlled RICFP, the possibility to achieve UV-induced polymerization of thick samples has been suggested. Mariani et al. reported this process for the first time [51]. The authors defined the proper photoinitiator–radical initiator ratio required to obtain a self-sustaining polymerization front. 

More recently, Bomze et al. described a new and more efficient system based on the same RICFP mechanism [52]. The authors polymerized the epoxy resin bisphenol-A-diglycidyl ether in the presence of a hexafluoroantimonate-based iodonium salt as photoacid generator and a benzopinacol as radical thermal initiator. In particular, it was shown that this system is characterized by a high storage stability and it was possible to achieve cross-linked epoxy material characterized by good mechanical properties. These findings open the idea that RICFP could be a powerful tool for the fast curing of epoxy composites; therefore, in a following paper, the effect of the presence of fillers dispersed into the same photocurable system was studied [53]. It was shown that the presence of SiO_2_ particles influenced the generation of a stable front. Due to the low thermal conductivity of the SiO_2_ particles, a delayed propagation of the front was observed. Although propagation was decelerated in the presence of fillers, a stable front was observed with a filler content up to 3 phr. In conclusion, successful UV-induced RICFP was also achieved for epoxy composites.

## 4. Conclusions

In conclusion, this review aims to show the uninterrupted importance and interest in the cationic photopolymerization process. Since its discovery at the end of the 70s by Prof. Crivello, the cationic photopolymerization process has met with continued interest in the scientific field, both in the synthesis of new and more efficient photoinitiators, able to expand the cationic process towards longer wavelength activation, and in new applications, such as the use of RICFP to prepare composites in a much more efficient way. This review is not intended to be comprehensive but to show new horizons for the cationic photopolymerization process. 

## Figures and Tables

**Table 1:** Porous iron(III)-based metal–organic frameworks (MOFs) employed as cationic promoted photoinitiators.

Organic linker	Terephthalic acid	Fumaric acid	Terephthalic acid	Azobenzene tetracarboxylic acid	Trimesic acid
	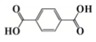	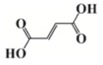	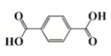	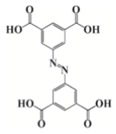	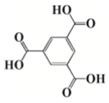
Cristalline structure	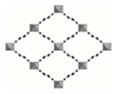	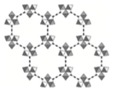	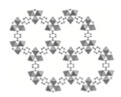	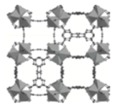	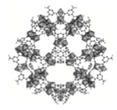
Chemical formula	Fe(OH)[C_8_O_4_H_4_] nsolv	Fe_3_O(OH)(H_2_O)_2_ [C_4_O_4_H_2_]_3_ nsolv	Fe_3_O(OH)(H_2_O)_2_ [C_8_O_4_H_4_]_3_ nsolv	Fe_3_O(OH)(H_2_O)_2_ [C_16_O_8_N_2_H_6_]_2_ nsolv	Fe_3_O(OH)(H_2_O)_2_ [C_9_O_6_H_3_]_2_ nsolv
%Fe	23.6 (chains)	30.9 (trimers)	24.2 (trimers)	21.4 (trimers)	25.8 (trimers)
Flexibility	Yes	Yes	Yes	No	No
Pore size (Å)	8.5	6.5	9	6 10	25 (5) 19 (8.6)
Particle size (µm)	2–3	5–9	0.06–0.10	0.5–0.7	0.1–0.4 0.05–0.10
